# Selecting manuscript for publication

**DOI:** 10.1186/s40902-016-0098-5

**Published:** 2016-12-05

**Authors:** Seong-Gon Kim

**Affiliations:** Department of Oral and Maxillofacial Surgery, College of Dentistry, Gangneung-Wonju National University, 7 Jukhyun-gil, Gangneung, 210-702 South Korea

## Editorial

*Maxillofacial Plastic and Reconstructive Surgery* (MPRS) is an official journal of the Korean Association of Maxillofacial Plastic and Reconstructive Surgeons (KAMPRS). It was launched at 1978 in the name of Journal of Korean Association of Maxillofacial Plastic and Reconstructive Surgeons. The journal title was changed to its current form in 2014 and published only articles written in English from that point onward. Since 2015, MPRS has been publishing approximately 50 articles a year in full open access format. Totally 30,919 times were recorded as a download number in 2015. Based on the analysis of countries, South Korea made up only 9% of the 30,919 downloads. Considering the increased number of open-access publications, the number of article downloads should be increased compared to 2015. The number of citations has increased sharply as well.

As the article processing charge is covered for KAMPRS members by the association, most submissions come from KAMPRS members. Currently, there are totally 2400 KAMPRS members. Now, KAMPRS also invites new members from out of South Korea. Some members are highly active authors, publishing more than five articles to international journals annually. The total number of articles published in top-ranking international journals, such as *Nature*, *Lancet*, and *Biomaterials*, has increased annually. Thus, the quality of articles submitted by KAMPRS members is not questionable. However, our association also has a wide spectrum of authors ranging from novice to professional. Particularly, the editorial board member finds many errors in the manuscripts written by trainees. For the board exam, two published articles are required. Thus, the submissions from trainees have not decreased but maintained annually.

Publishing highly cited article is a major interest for editor. Among the articles published in 2015, several articles have not been cited. Examples of these include “For arthrocentesis, ultra-thin arthroscopy can be used” [1] and “For the palatal expansion, custom-made appliances can be considered” [2]. Though some issue raised in these articles may be interesting, novel findings are few. As increasing competition for publication, there will be little chance to see these types of article in the future. By the way, the number of citations is not always associated with readership. For example, “The condylar position after unilateral sagittal split ramus osteotomy” [3] has rarely been published. The download number of this article was actually 6043 when searched on September 28, 2016. This article is ranked first in terms of the number of downloads among published articles in MPRS since 2015, but it has not been cited until now. The reason may be that this article handles a very uncommon situation. At any rate, this issue should be interesting to the readers of MPRS.

Selecting proper articles for publication is not easy. Although original articles have a higher priority for publication, case reports should also be considered for publication. Regardless of citation matters, the editors should consider publishing interesting articles, such as Kim et al.’s paper [3]. Citation can be increased by adopting data sharing policy. From 2017, MPRS will adopt data sharing policy. The authors who consider publishing their article to MPRS should consider the data sharing policy. Detailed policy will be announced in the web page.
